# Supplementary material for: SBMLDiagrams: a python package to process and visualize SBML layout and render
Source: Bioinformatics. 2022 Nov 12;39(1):btac730. doi: 10.1093/bioinformatics/btac730 (PMC9805581; doi:10.1093/bioinformatics/btac730)
Supplement: btac730_Supplementary_Data [file btac730_supplementary_data.pdf]

Supplementary Information

SBMLDiagrams: A python package to process and visualize SBML layout and render

Jin Xu [1], Jessie Jiang [2] and Herbert M. Sauro [1]

1Department of Bioengineering, University of Washington, Seattle, 98195, USA and
2Department of Computer Science and Engineering, University of Washington, Seattle, 98195, USA

Examples to compare the use of libSBML and SBMLDiagrams

To read and write SBML, users can use the software library libSBML. However, it can be difficult for new users to learn python-libSBML which requires a detailed knowledge of the layout and render object model. In the following, three simple examples illustrate how SBMLDiagrams can make reading SBML files significantly easier and more convenient than using python-libSBML.

To compare reading SBML files by SBMLDiagrams and python-libSBML, there is a separate python script with three simple functions, i.e. getNodeSize(), getNodeBorderWidth() and getNodeBorderColor(). The script is available on GitHub (https://github.com/sys-bio/SBMLDiagrams/blob/main/examples/supplementary/supplementary_Compare_libSBML.py). When using SBMLDiagrams, it only takes one line to load SBML string and one additional line for each function: getNodeSize(), getNodeBorderWidth() and getNodeBorderColor(). However, it takes over one-hundred lines to achieve the same outcome when using python-libSBML with two classes and four functions.

First, it is necessary to judge the validity of an imported SBML string and check whether there is the layout and/or render information. Secondly, it is critical to read the layout and render information separately from the SBML string. Finally, there are supposed to be additional warnings and support to cross check users' inputs and help with organizing the information read out from SBML files.

Example 1: getNodeSize()

First, all the input arguments are supposed to get validated, including the SBML string, node id, and alias node index. If there is an error, a corresponding warning will get raised. Second, the information of node size can be obtained from the layout, which is stored as the width and height values from the bounding box of each species glyph. Finally, the output of the node size is written as a Point object to make it more applicable. When using SBMLDiagrams, it only takes one line to load the SBML string and one additional line for the function of getNodeSize().

However, it takes about 50 lines to achieve the same function via python-libSBML. The script to compare SBMLDiagrams and libSBML for getNodeSize() is below and also available on GitHub (https://github.com/sys-bio/SBMLDiagrams/blob/main/examples/supplementary/supplementary_Compare_libSBML_getNodeSize.py)

Script using SBMLDiagrams (3 lines)

```
import SBMLDiagrams

sd = SBMLDiagrams.load(sbmlStr)

print("Get node size by SBMLDiagrams (width, hight):", sd.getNodeSize("x_0").x, sd.getNodeSize("x_0").y)
```

Script using libSBML (~50 lines excluding comments)

```
import libsbml

class Point:
    def __init__(self,x_init=0,y_init=0):
        """
        Define a Point object with attributes x and y.

        """
        self.x = x_init
        self.y = y_init

class Load:
    def __init__(self, sbmlStr = ''):
        """
        Load the SBML string.

        Args:
            sbmlStr: str-the SBML string.
        """
        self.sbmlStr = sbmlStr

        self.spec_specGlyph_id_list = []
        self.spec_dimension_list = []

        mplugin = None
        try:
            #check the validity of the sbml files.
            document = libsbml.readSBMLFromString(self.sbmlStr)
            if document.getNumErrors() != 0:
                errMsgRead = document.getErrorLog().toString()
                raise Exception("Errors in SBML Model: ", errMsgRead)
            ### from here for layout ###
            model_layout = document.getModel()
            try:
                mplugin = model_layout.getPlugin("layout")
```

```
            except:
                raise Exception("There is no layout.")
            if mplugin is not None:
                layout = mplugin.getLayout(0)
                if layout is not None:
                    self.numSpecGlyphs = layout.getNumSpeciesGlyphs()
                    for i in range(self.numSpecGlyphs):
                        specGlyph = layout.getSpeciesGlyph(i)
                        specGlyph_id = specGlyph.getId()
                        spec_id = specGlyph.getSpeciesId()
                        self.spec_specGlyph_id_list.append([spec_id,specGlyph_id])
                        boundingbox = specGlyph.getBoundingBox()
                        height = boundingbox.getHeight()
                        width = boundingbox.getWidth()
                        self.spec_dimension_list.append([width,height])

        except Exception as e:
            raise Exception (e)

    def getNodeSize(self, id, alias = 0):
        """
        Get the size of a node with a given node id.

        Args:
            id: str-the id of the node.

            alias: int-alias node index: 0 to number of alias nodes -1.

        Returns:
            size: a Point object with attributes x and y representing
            the width and height of the node.

        Examples:
            p =  Load(sbmlStr).getNodeSize('ATP')

            print ('Width = ', p.x, 'Height = ', p.y)

        """
        size_list = []
        for i in range(self.numSpecGlyphs):
            if id == self.spec_specGlyph_id_list[i][0]:
                s = self.spec_dimension_list[i]
                size = Point(s[0], s[1])
                size_list.append(size)

        if len(size_list) == 0:
            raise Exception("This is not a valid id.")
        if alias < len(size_list) and alias >= 0:
            return size_list[alias]
        else:
            raise Exception("Alias index is beyond number of alias nodes.")

ls = Load(sbmlStr)
print("Get node size by libSBML (width, height):", ls.getNodeSize("x_0").x, ls.getNodeSize("x_0").y)
```

Example 2: getNodeBorderWidth()

The first step is to check the validity of inputs and set up warnings as necessary. The node border width can be read from render, which is stored as the stroke width information from each specific species glyph's style. When using SBMLDiagrams, it only takes one line to load the SBML string and one additional line for the function of getNodeBorderWidth(). However, it takes about 50 lines to achieve the same function via python-libSBML. The script to compare SBMLDiagrams and libSBML for getNodeBorderWidth() is below and also available on GitHub (https://github.com/sys-bio/SBMLDiagrams/blob/main/examples/supplementary/supplementary_Compare_libSBML_getNodeBorderWidth.py)

Script using SBMLDiagrams (3 lines)

```
import SBMLDiagrams

sd = SBMLDiagrams.load(sbmlStr)

print("Get node border width by SBMLDiagrams:", sd.getNodeBorderWidth("x_0"))
```

Script using libSBML (~50 lines excluding comments)

```
import libsbml

class Load:
    def __init__(self, sbmlStr = ''):
        """
        Load the SBML string.

        Args:
            sbmlStr: str-the SBML string.
        """
        self.sbmlStr = sbmlStr

        self.spec_render = []

        mplugin = None
        try:
            #check the validity of the sbml files.
            document = libsbml.readSBMLFromString(self.sbmlStr)
            if document.getNumErrors() != 0:
                errMsgRead = document.getErrorLog().toString()
                raise Exception("Errors in SBML Model: ", errMsgRead)
            ### from here for layout ###
            model_layout = document.getModel()
            try:
                mplugin = model_layout.getPlugin("layout")
            except:
                raise Exception("There is no layout.")
```

```
            if mplugin is not None:
                layout = mplugin.getLayout(0)
                if layout is not None:
                    ### from here for render ###
                    rPlugin = layout.getPlugin("render")
                    if (rPlugin != None and rPlugin.getNumLocalRenderInformationObjects() > 0):
                        info = rPlugin.getRenderInformation(0)
                        for j in range (0, info.getNumStyles()):
                            style = info.getStyle(j)
                            group = style.getGroup()
                            typeList = style.createTypeString()
                            idList = style.createIdString()
                            if 'SPECIESGLYPH' in typeList:
                                spec_border_width = group.getStrokeWidth()
                                self.spec_render.append([idList, spec_border_width])

        except Exception as e:
            raise Exception (e)


    def getNodeBorderWidth(self, id, alias = 0):
        """
        Get the border width of a node with a given node id.

        Args:
            id: str-the id of the node.

            alias: int-alias node index: 0 to number of alias nodes -1.

        Returns:
            border_width: float-node border line width.

        """

        border_width_list = []
        for i in range(len(self.spec_render)):
            if id == self.spec_render[i][0]:
                spec_border_width = self.spec_render[i][1]
                border_width_list.append(spec_border_width)

        if len(border_width_list) == 0:
            raise Exception("This is not a valid id.")
        if alias < len(border_width_list) and alias >= 0:
            return border_width_list[alias]
        else:
            raise Exception("Alias index is beyond number of alias nodes.")

ls = Load(sbmlStr)
print("Get node border width by libSBML:", ls.getNodeBorderWidth("x_0"))
```

Example3: getNodeBorderColor()

After checking the validity of inputs with warnings, the main step is to read the border color from the render extension. The node border color is the stroke information inside each specific

species glyph's style. In addition, the color information stored in an SBML file is a HEX string, which needs to be changed to a more human readable format as a decimal RGB(A) matrix or an HTML name if applicable. When using SBMLDiagrams, it only takes one line to load the SBML string and one additional line for the function of getNodeBorderColor(). However, it takes about 90 lines to achieve the same function via python-libSBML. The script to compare SBMLDiagrams and libSBML for getNodeBorderColor() is below and also available on GitHub (https://github.com/sys-bio/SBMLDiagrams/blob/main/examples/supplementary/supplementary_Compare_libSBML_getNodeBorderColor.py).

Script using SBMLDiagrams (3 lines)

```
import SBMLDiagrams

sd = SBMLDiagrams.load(sbmlStr)

print("Get node border color by SBMLDiagrams:", sd.getNodeBorderColor("x_0"))
```

Show script using libSBML (~90 lines excluding comments)

```
import libsbml
import pandas as pd

class Load:
    def __init__(self, sbmlStr = ''):
        """
        Load the SBML string.

        Args:
            sbmlStr: str-the SBML string.
        """
        self.sbmlStr = sbmlStr

        def hex_to_rgb(value):
            """
            Change color format from hex string to rgb.
            """
            value = value.lstrip('#')
            if len(value) == 6:
                value = value + 'ff'
            return [int(value[i:i+2], 16) for i in (0, 2, 4, 6)]

        self.spec_render = []

        mplugin = None
        try:
            #check the validity of the sbml files.
            document = libsbml.readSBMLFromString(self.sbmlStr)
            if document.getNumErrors() != 0:
```

```
            errMsgRead = document.getErrorLog().toString()
            raise Exception("Errors in SBML Model: ", errMsgRead)
        ### from here for layout ###
        model_layout = document.getModel()
        try:
            mplugin = model_layout.getPlugin("layout")
        except:
            raise Exception("There is no layout.")
        if mplugin is not None:
            layout = mplugin.getLayout(0)
            if layout is not None:
                rPlugin = layout.getPlugin("render")
                if (rPlugin != None and rPlugin.getNumLocalRenderInformationObjects() > 0):
                    info = rPlugin.getRenderInformation(0)
                    color_list = []
                    for  j in range(0, info.getNumColorDefinitions()):
                        color = info.getColorDefinition(j)
                        color_list.append([color.getId(),color.createValueString()])
                    for j in range (0, info.getNumStyles()):
                        style = info.getStyle(j)
                        group = style.getGroup()
                        typeList = style.createTypeString()
                        idList = style.createIdString()
                        if 'SPECIESGLYPH' in typeList:
                            for k in range(len(color_list)):
                                if color_list[k][0] == group.getStroke():
                                    spec_border_color = hex_to_rgb(color_list[k][1])
                            self.spec_render.append([idList, spec_border_color])

    except Exception as e:
        raise Exception (e)

def getNodeBorderColor(self, id, alias = 0):
    """
    Get the border width of a node with a given node id.

    Args:
        id: str-the id of the node.

        alias: int-alias node index: 0 to number of alias nodes -1.

    Returns:
        border_color: list-[rgba 1*4 matrix, html_name str (if any, otherwise ''),
        hex str (8 digits)].

    """
    color_data = {"decimal_rgb": ['[240,248,255]', '[250,235,215]', '[0,255,255]', '[127,255,212]', '[240,255,255]',
'[245,245,220]', '[255,228,196]', '[0,0,0]', '[255,235,205]', '[0,0,255]', '[138,43,226]', '[165,42,42]', '[222,184,135]', '[95,158,160]',
'[127,255,0]', '[210,105,30]', '[255,127,80]', '[100,149,237]', '[255,248,220]', '[220,20,60]', '[0,255,255]', '[0,0,139]', '[0,139,139]',
'[184,134,11]', '[169,169,169]', '[0,100,0]', '[189,183,107]', '[139,0,139]', '[85,107,47]', '[255,140,0]', '[153,50,204]', '[139,0,0]',
'[233,150,122]', '[143,188,143]', '[72,61,139]', '[47,79,79]', '[0,206,209]', '[148,0,211]', '[255,20,147]', '[0,191,255]',
'[105,105,105]', '[30,144,255]', '[178,34,34]', '[255,250,240]', '[34,139,34]', '[255,0,255]', '[220,220,220]', '[248,248,255]',
'[255,215,0]', '[218,165,32]', '[128,128,128]', '[0,128,0]', '[173,255,47]', '[240,255,240]', '[255,105,180]', '[205,92,92]', '[75,0,130]',
'[255,255,240]', '[240,230,140]', '[230,230,250]', '[255,240,245]', '[124,252,0]', '[255,250,205]', '[173,216,230]', '[240,128,128]',
```

```
'[224,255,255]', '[250,250,210]', '[144,238,144]', '[211,211,211]', '[255,182,193]', '[255,160,122]', '[32,178,170]', '[135,206,250]',
'[119,136,153]', '[176,196,222]', '[255,255,224]', '[0,255,0]', '[50,205,50]', '[250,240,230]', '[255,0,255]', '[128,0,0]',
'[102,205,170]', '[0,0,205]', '[186,85,211]', '[147,112,219]', '[60,179,113]', '[123,104,238]', '[0,250,154]', '[72,209,204]',
'[199,21,133]', '[25,25,112]', '[245,255,250]', '[255,228,225]', '[255,228,181]', '[255,222,173]', '[0,0,128]', '[253,245,230]',
'[128,128,0]', '[107,142,35]', '[255,165,0]', '[255,69,0]', '[218,112,214]', '[238,232,170]', '[152,251,152]', '[175,238,238]',
'[219,112,147]', '[255,239,213]', '[255,218,185]', '[205,133,63]', '[255,192,203]', '[221,160,221]', '[176,224,230]', '[128,0,128]',
'[255,0,0]', '[188,143,143]', '[65,105,225]', '[139,69,19]', '[250,128,114]', '[244,164,96]', '[46,139,87]', '[255,245,238]',
'[160,82,45]', '[192,192,192]', '[135,206,235]', '[106,90,205]', '[112,128,144]', '[255,250,250]', '[0,255,127]', '[70,130,180]',
'[210,180,140]', '[0,128,128]', '[216,191,216]', '[255,99,71]', '[64,224,208]', '[238,130,238]', '[245,222,179]', '[255,255,255]',
'[245,245,245]', '[255,255,0]', '[154,205,50]'],\
    "html_name":['AliceBlue', 'AntiqueWhite', 'Aqua', 'Aquamarine', 'Azure', 'Beige', 'Bisque', 'Black', 'BlanchedAlmond', 'Blue',
'BlueViolet', 'Brown', 'BurlyWood', 'CadetBlue', 'Chartreuse', 'Chocolate', 'Coral', 'CornflowerBlue', 'Cornsilk', 'Crimson', 'Cyan',
'DarkBlue', 'DarkCyan', 'DarkGoldenrod', 'DarkGray', 'DarkGreen', 'DarkKhaki', 'DarkMagenta', 'DarkOliveGreen', 'DarkOrange',
'DarkOrchid', 'DarkRed', 'DarkSalmon', 'DarkSeaGreen', 'DarkSlateBlue', 'DarkSlateGray', 'DarkTurquoise', 'DarkViolet',
'DeepPink', 'DeepSkyBlue', 'DimGray', 'DodgerBlue', 'FireBrick', 'FloralWhite', 'ForestGreen', 'Fuchsia', 'Gainsboro',
'GhostWhite', 'Gold', 'Goldenrod', 'Gray', 'Green', 'GreenYellow', 'Honeydew', 'HotPink', 'IndianRed', 'Indigo', 'Ivory', 'Khaki',
'Lavender', 'LavenderBlush', 'LawnGreen', 'LemonChiffon', 'LightBlue', 'LightCoral', 'LightCyan', 'LightGoldenrodYellow',
'LightGreen', 'LightGrey', 'LightPink', 'LightSalmon', 'LightSeaGreen', 'LightSkyBlue', 'LightSlateGray', 'LightSteelBlue',
'LightYellow', 'Lime', 'LimeGreen', 'Linen', 'Magenta', 'Maroon', 'MediumAquamarine', 'MediumBlue', 'MediumOrchid',
'MediumPurple', 'MediumSeaGreen', 'MediumSlateBlue', 'MediumSpringGreen', 'MediumTurquoise', 'MediumVioletRed',
'MidnightBlue', 'MintCream', 'MistyRose', 'Moccasin', 'NavajoWhite', 'Navy', 'OldLace', 'Olive', 'OliveDrab', 'Orange',
'OrangeRed', 'Orchid', 'PaleGoldenrod', 'PaleGreen', 'PaleTurquoise', 'PaleVioletRed', 'PapayaWhip', 'PeachPuff', 'Peru', 'Pink',
'Plum', 'PowderBlue', 'Purple', 'Red', 'RosyBrown', 'RoyalBlue', 'SaddleBrown', 'Salmon', 'SandyBrown', 'SeaGreen', 'Seashell',
'Sienna', 'Silver', 'SkyBlue', 'SlateBlue', 'SlateGray', 'Snow', 'SpringGreen', 'SteelBlue', 'Tan', 'Teal', 'Thistle', 'Tomato', 'Turquoise',
'Violet', 'Wheat', 'White', 'WhiteSmoke', 'Yellow', 'YellowGreen'],\
    "hex_string":['#F0F8FF', '#FAEBD7', '#00FFFF', '#7FFFD4', '#F0FFFF', '#F5F5DC', '#FFE4C4', '#000000', '#FFEBCD',
'#0000FF', '#8A2BE2', '#A52A2A', '#DEB887', '#5F9EA0', '#7FFF00', '#D2691E', '#FF7F50', '#6495ED', '#FFF8DC', '#DC143C',
'#00FFFF', '#00008B', '#008B8B', '#B8860B', '#A9A9A9', '#006400', '#BDB76B', '#8B008B', '#556B2F', '#FF8C00', '#9932CC',
'#8B0000', '#E9967A', '#8FBC8F', '#483D8B', '#2F4F4F', '#00CED1', '#9400D3', '#FF1493', '#00BFFF', '#696969', '#1E90FF',
'#B22222', '#FFFAF0', '#228B22', '#FF00FF', '#DCDCDC', '#F8F8FF', '#FFD700', '#DAA520', '#808080', '#008000', '#ADFF2F',
'#F0FFF0', '#FF69B4', '#CD5C5C', '#4B0082', '#FFFFF0', '#F0E68C', '#E6E6FA', '#FFF0F5', '#7CFC00', '#FFFACD',
'#ADD8E6', '#F08080', '#E0FFFF', '#FAFAD2', '#90EE90', '#D3D3D3', '#FFB6C1', '#FFA07A', '#20B2AA', '#87CEFA',
'#778899', '#B0C4DE', '#FFFFE0', '#00FF00', '#32CD32', '#FAF0E6', '#FF00FF', '#800000', '#66CDAA', '#0000CD', '#BA55D3',
'#9370DB', '#3CB371', '#7B68EE', '#00FA9A', '#48D1CC', '#C71585', '#191970', '#F5FFFA', '#FFE4E1', '#FFE4B5',
'#FFDEAD', '#000080', '#FDF5E6', '#808000', '#6B8E23', '#FFA500', '#FF4500', '#DA70D6', '#EEE8AA', '#98FB98',
'#AFEEEE', '#DB7093', '#FFEFD5', '#FFDAB9', '#CD853F', '#FFC0CB', '#DDA0DD', '#B0E0E6', '#800080', '#FF0000',
'#BC8F8F', '#4169E1', '#8B4513', '#FA8072', '#F4A460', '#2E8B57', '#FFF5EE', '#A0522D', '#C0C0C0', '#87CEEB', '#6A5ACD',
'#708090', '#FFFAFA', '#00FF7F', '#4682B4', '#D2B48C', '#008080', '#D8BFD8', '#FF6347', '40E0D0', '#EE82EE', '#F5DEB3',
'#FFFFFF', '#F5F5F5', '#FFFF00', '#9ACD32']}
    df_color = pd.DataFrame(color_data)

    def _rgb_to_color(rgb):
        """
        transfer a list of rgb to a color list with decimal_rgba, html_name and hex_string.

        Args:
            rgb: list-1*3 or 1*4 matrix for a decimal rgb or rgba.

        Returns:
            color: list-[decimal_rgb, html_name, hex_string].

        """
        color = []
        if len(rgb) == 3:
```

```
            #decial_rgba:
            rgba = rgb.copy()
            a = 255 # default is fully opaque, the value should be int 255.
            rgba.append(a)
            #hex_string:
            hex_str = '#%02X%02X%02X%02X' % (rgba[0],rgba[1],rgba[2],rgba[3])
            #html_name:
            html_name = ''
            hex_str_search = hex_str[0:-2]
            if hex_str_search in df_color.values:
                index = df_color.index[df_color["hex_string"] == hex_str_search].tolist()[0] #row index
                html_name = df_color.iloc[index]["html_name"]
            color.append(rgba)
            color.append(html_name)
            color.append(hex_str)
        elif len(rgb) == 4:
            #decial_rgba:
            rgba = rgb.copy()
            #hex_string:
            hex_str = '#%02X%02X%02X%02X' % (rgba[0],rgba[1],rgba[2],rgba[3])
            #html_name:
            html_name = ''
            hex_str_search = hex_str[0:-2]
            if hex_str_search in df_color.values:
                index = df_color.index[df_color["hex_string"] == hex_str_search].tolist()[0] #row index
                html_name = df_color.iloc[index]["html_name"]
            color.append(rgba)
            color.append(html_name)
            color.append(hex_str)
        else:
            color = [[], '', '']

        return color

    border_color_list = []
    for i in range(len(self.spec_render)):
        if id == self.spec_render[i][0]:
            rgb = self.spec_render[i][1]
            color = _rgb_to_color(rgb)
            border_color_list.append(color)

    if len(border_color_list) == 0:
        raise Exception("This is not a valid id.")
    if alias < len(border_color_list) and alias >= 0:
        return border_color_list[alias]
    else:
        raise Exception("Alias index is beyond number of alias nodes.")

ls = Load(sbmlStr)
print("Get node border color by libSBML:", ls.getNodeBorderColor("x_0"))
```

Test suite for testing support of SBML layout and render

In order to test SBMLDiagrams support for SBML layout and render, we have prepared a test suite to cover the possible features. All the SBML files and figures generated are available on GitHub (https://github.com/sys-bio/SBMLDiagrams/tree/main/SBMLDiagrams/test_sbml_files/test_suite).

Test 1: BIOMD0000000005.xml

This SBML file is from CellDesigner (Funahashi *et al.*, 2006) which uses its own proprietary format, and does not use the standard layout and render extensions. SBMLDiagrams can visualize it by randomly positioning the nodes when layout information is not given. The random positioning will be different each time when the model is imported. However, users can generate an SBML file with the layout and render by SBMLDiagrams. The layout and render information can be saved in the exported SBML file (BIOMD0000000005_layout_render.xml) by the export() function. Then, users can obtain the same figure by importing the generated file via the load() function and visualizing it by the draw() function.

Test 2: Carcione2020.xml

This is the SBML file of the deterministic SEIR simulation of a COVID-19 outbreak (Carcione *et al.*, 2020). The node texts can be in different font colors.

Test 3: Garde2020.xml

This is the SBML file of the minimal model of metabolic oscillations in *Bacillus subtilis* biofilms (Garde *et al.*, 2020). There are no reactants or products in some reactions. The reaction centroids are in the shape of circles with texts of "R" in green.

Test 4: Jana_WolfGlycolysis.xml

This is an SBML file from a model of glycolysis (Wolf *et al.*, 2001). It is an example with alias nodes and long text. The nodes with borders in dashed lines are alias nodes. The nodes with borders with thicker lines are boundary nodes. The long text is fitted to the size of the node with automatically decreasing the text font size.

Test 5: LinearChain.xml

This SBML file is an example with the layout but without the render extension. SBMLDiagrams positions the nodes according to the layout information and assigns the default render information automatically.

Test 6: feedback.xml

This SBML file is an example without the layout and render extensions. In addition, there is a modifier in the model. SBMLDiagrams can assign a random position and default render to it.

Test 7: global_render.xml
This SBML file is generated by COPASI (Hopps *et al.*, 2007). It is an example of an SBML file with global render, i.e. node fill color and border color, reaction line color, etc.

Test 8: mass_action_rxn.xml
This SBML file is an example with Bezier reactions and different reaction line colors.

Test 9: node_grid.xml
This SBML file is an example with orphan nodes without reactions. Nodes are in different shapes with various fill and border colors.

Test 10: pdmap-nucleoid.xml
This SBML file is generated by MINERVA (Gawron *et al.*, 2016). It is an example of an SBML file with complex nodes, dashed reaction lines, reactions with different arrowheads, modifiers with different shapes and nodes with different text anchors.

Test 11: sbml_error/testbigmodel.xml
This is an SBML file with errors. SBMLDiagrams will alert the user with appropriate error messages.

Test 12: test.xml
This is an SBML file with both layout and render information. It is a simple case with two nodes and one reaction.

Test 13: test_centroid.xml
This is an SBML file with a centroid in the shape of a circle with a certain fill and border color as part of the reaction.

Test 14: test_comp.xml
This is an SBML with compartments in different colors. Nodes are in different shapes and border colors. Reaction lines are in different colors. Alias nodes and boundary nodes are shown by dashed border lines and thicker border lines separately.

Test 15: test_genGlyph.xml
This SBML file has only one rectangle to indicate that SBMLDiagrams can support arbitrary shapes as GENERALGLYPH in the render.

Test 16: test_gradientLinear.xml

This is an SBML file with only one node filled with linear gradients from the left (white) to the right (silver).

Test 17: test_gradientRadial.xml
This is an SBML file with only one node filled with radial gradients from the center (white) to the node border (silver).

Test 18: test_modifier.xml
This is an SBML file with a modifier given layout and render information.

Test 19; test_no_comp.xml
This is an SBML file with node texts positioned outside the nodes. Nodes and reaction lines in different colors are also shown.

Test 20: test_textGlyph.xml
This is an SBML file with texts only to indicate that SBMLDiagrams can support arbitrary texts as TEXTGLYPH in the render.

References

Carcione J.M., Santos J.E., Bagaini C., Ba J. (2020) A Simulation of a COVID-19 Epidemic Based on a Deterministic SEIR Model. *Front. Public Health* **8**, 230.

Funahashi A., Matsuoka Y., Jouraku A., Kitano H., Kikuchi, N. (2006). CellDesigner: a modeling tool for biochemical networks. WSC '06: Proceedings of the 38th conference on Winter simulation.

Garde R., Ibrahim B., Schuster S. (2020). Extending the minimal model of metabolic oscillations in *Bacillus subtilis* biofilms. *Sci Rep* **10**, 5579.

Gawron P., Ostaszewski M., Satagopam V. *et al.* (2016). MINERVA—a platform for visualization and curation of molecular interaction networks. *npj Syst Biol Appl* **2**, 16020.

Hoops S., Sahle S., Gauges R. *et al.* (2007). COPASI—a complex pathway simulator. *Bioinformatics*, **22**(24). 3067-74.

Wolf J., Sohn H.-Y., Heinrich R., Kuriyama H. (2001). Mathematical analysis of a mechanism for autonomous metabolic oscillations in continuous culture of Saccharomyces cerevisiae. *FEBS Lett*, **499**(3), 230-234.
